# Genetic determinants of serum bilirubin using inferred native American gene variants in Chilean adolescents

**DOI:** 10.3389/fgene.2024.1382103

**Published:** 2024-05-17

**Authors:** José P. Miranda, Ana Pereira, Camila Corvalán, Juan F. Miquel, Gigliola Alberti, Juan C. Gana, José L. Santos

**Affiliations:** ^1^ Department of Nutrition, Diabetes, and Metabolism, School of Medicine, Pontificia Universidad Católica de Chile, Santiago, Chile; ^2^ School of Medicine, PhD in Epidemiology Program, Pontificia Universidad Católica de Chile, Santiago, Chile; ^3^ Advanced Center for Chronic Diseases (ACCDiS), Pontificia Universidad Católica de Chile and Universidad de Chile, Santiago, Chile; ^4^ Instituto de Nutrición y Tecnología de los Alimentos INTA, Universidad de Chile, Santiago, Chile; ^5^ Department of Gastroenterology, School of Medicine, Pontificia Universidad Católica de Chile, Santiago, Chile; ^6^ Pediatrics Division, School of Medicine, Pontificia Universidad Católica de Chile, Santiago, Chile; ^7^ Department of Gastroenterology and Pediatric Nutrition, School of Medicine, Pontificia Universidad Católica de Chile, Santiago, Chile

**Keywords:** bilirubin, local ancestry deconvolution, GWAS, population genomics, genetic epidemiology, native American, UGT1A1

## Abstract

Gene variants in the *UGT1A1* gene are strongly associated with circulating bilirubin levels in several populations, as well as other variants of modest effect across the genome. However, the effects of such variants are unknown regarding the Native American ancestry of the admixed Latino population. Our objective was to assess the Native American genetic determinants of serum bilirubin in Chilean admixed adolescents using the local ancestry deconvolution approach. We measured total serum bilirubin levels in 707 adolescents of the Chilean Growth and Obesity Cohort Study (GOCS) and performed high-density genotyping using the Illumina-MEGA array (>1.7 million genotypes). We constructed a local ancestry reference panel with participants from the 1000 Genomes Project, the Human Genome Diversity Project, and our GOCS cohort. Then, we inferred and isolated haplotype tracts of Native American, European, or African origin to perform genome-wide association studies. In the whole cohort, the rs887829 variant and others near *UGT1A1* were the unique signals achieving genome-wide statistical significance (b = 0.30; *p* = 3.34 × 10^−57^). After applying deconvolution methods, we found that significance is also maintained in Native American (b = 0.35; *p* = 3.29 × 10^−17^) and European (b = 0.28; *p* = 1.14 × 10^−23^) ancestry components. The rs887829 variant explained a higher percentage of the variance of bilirubin in the Native American (37.6%) compared to European ancestry (28.4%). In Native American ancestry, carriers of the TT genotype of this variant averaged 4-fold higher bilirubinemia compared to the CC genotype (*p* = 2.82 × 10^−12^). We showed for the first time that *UGT1A1* variants are the primary determinant of bilirubin levels in Native American ancestry, confirming its pan-ethnic relevance. Our study illustrates the general value of the local ancestry deconvolution approach to assessing isolated ancestry effects in admixed populations.

## Introduction

Bilirubin is the end-product of heme degradation, mainly derived from erythrocytic hemoglobin (−80% of total bilirubin production) and, to a lesser extent, from other hemoproteins such as myoglobin, cytochromes, and catalases. After roughly 120 days of the erythrocyte functional life, macrophages of mononuclear phagocyte system of the spleen, bone marrow, and Kupfer cells of the liver engulf senescent erythrocytes and degrade them to release the prosthetic heme group from globin chains. The rate-limiting enzyme of heme catabolism is heme oxygenase (HMOX). This enzyme opens the porphyrin ring to generate Fe2+, CO, and the green pigment biliverdin, consuming NADPH. After the HMOX reaction, biliverdin is degraded by biliverdin reductase using NADPH to produce bilirubin, a lipid-soluble compound of yellowish-orange color. In circulation, bilirubin is bound to albumin and taken into the liver by members of the human organic anion-transporting polypeptide (OATP) family (Fujiwara et al., 2017). Once in the liver, bilirubin is conjugated with glucuronic acid (mono- and, mostly, diglucuronide bilirubin) exclusively by the UDP-glucuronosyltransferase 1A1 (UGT1A1) enzyme (Ritter et al., 1991). The conjugated bilirubin (also called “direct bilirubin”) is a water-soluble compound that is transported into bile via the ATP-dependent multidrug-resistant protein transporter MRP2 (*ABCC2*) in the canalicular hepatocyte membrane (Jedlitschky et al., 1997).

Total circulating bilirubin (TB) levels (typically in the range of 0.2–1.0 mg/dL) are determined by the rate of enzymes involved in heme synthesis and degradation, liver uptake, glucuronidation, hepatic excretion, absorption from the gut and transport. During a lifetime, circulating TB levels are reported to increase during childhood and adolescence, reaching a peak between 25–30 years and then decreasing with age. TB levels are higher in men than women and among nonsmokers than smokers. Additionally, ethnic differences are reported concerning higher levels among Mexican Americans compared to non-Hispanic blacks (Rosenthal et al., 1984; Zucker et al., 2004). From a physiopathology perspective, hyperbilirubinemia is classified according to whether it is due to an increased bilirubin production by enhanced hemolysis; defects in conjugation caused by severe mutations in *UGT1A1* causing Crigler-Najjar type I and II syndromes; due to a hepatocellular alteration, as in the case of viral hepatitis, some autoimmune liver diseases, or by biliary atresia, as it occurs in obstructions by gallstones or tumors (Méndez-Sánchez et al., 2019). A mild increase of serum bilirubin levels is also observed in the Gilbert syndrome, a benign condition caused by a TA-insertion in the TATA box of *UGT1A1* promoter [A (TA)_7_TAA] (termed UGT1A1*28; instead of the normal TA_6_), leading to 50%–70% reduction in *UGT1A1* gene expression (Strassburg, 2010).

At a population level, several genome-wide association studies (GWAS) have identified the *UGT1A* on chromosome 2q37.1 as the major locus influencing serum bilirubin, including European (Johnson et al., 2009; Sanna et al., 2009), Australian (Benton et al., 2015), African American (Chen et al., 2013), and Asian (Kang et al., 2010; Dai et al., 2013) populations. Among Europeans, there are also other significant variants modestly associated with bilirubin levels in the solute carrier organic anion transporter family member 1B1, 1B3, and 1B7 genes (*SLCO1B1*, *SLCO1B3,* and *SLCO1B7*), which encode for a membrane-bound sodium-independent organic anion transporter (Johnson et al., 2009; Sanna et al., 2009; Sakaue et al., 2021; Sinnott-Armstrong et al., 2021). However, little is known about Native American ancestry at a genome-wide scale (Kronenberg et al., 2002; Lin et al., 2009a; Melton et al., 2011). Assessing the Native American ancestry in genetic studies is important since Latino populations (a genetic admixture of Native American, European, and African origins) (Eyheramendy et al., 2015; Chacón-Duque et al., 2018) have been largely excluded from such studies. However, assessing the effect of gene variants on phenotypes in Native American ancestry is difficult given the lack of cohorts with participants of this ancestry because this population began a process of admixing with European and African populations just over 500 years ago. To overcome this limitation, a new strategy was recently reported that allows the determination of each variant’s ancestry locally, using the local ancestry deconvolution approach (Atkinson et al., 2021). This method generates multiple sub-cohorts within an admixed population to disentangle an isolated ancestral component by extracting and tagging haplotype tracts. Then, our study aimed to perform a Local Ancestry Deconvoluted GWAS (LAD-GWAS) to evaluate the isolated effect of inferred Native American ancestry on serum bilirubin regulation in Chilean adolescents.

## Subjects and methods

### Study design

**FIGURE 1:**
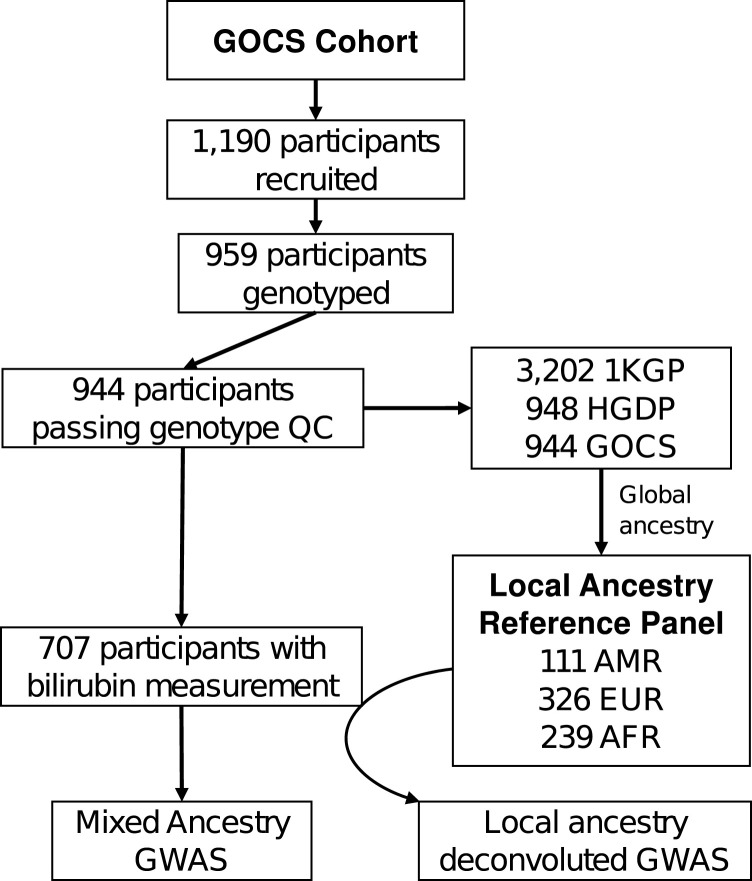
Flow analysis in GOCS cohort for the admixed ancestry GWAS or Local Ancestry Deconvoluted GWAS of bilirubin. 1KGP and HGDP correspond to the genotypes of the 1000 Genomes Project and the Human Genome Diversity Project participants, respectively. AMR: admixed Latino population, EUR: European ancestry, AFR: African ancestry.

We evaluated adolescents enrolled in the Growth and Obesity Chilean Cohort Study (GOCS). This cohort was created in 2006 and follows 1,190 children aged 2.6–4.0 years at enrollment who attended public nursery schools of the Chilean National Preschool Program (JUNJI) as well as the Chilean National School Board Program (JUNAEB) in six counties in Santiago de Chile (Sánchez et al., 2016). We genotyped a subset of 964 participants by microarray and measured serum bilirubin levels on 707 adolescents at age 15.4 ± 0.98 years (341 males, 366 females). A diagram of the analysis flow is shown in Figure 1.

This study was approved by the Ethics Review Board of the School of Medicine (Pontificia Universidad Católica de Chile). Written informed consent was obtained from the parents or guardians of each participant of the GOCS cohort.

### Anthropometric and biochemical measurements

The Body Mass Index (BMI) for age z-score (BAZ) was measured according to WHO recommendations (De Onis et al., 2007). Using the Diazo method, we measured TB on a Cobas C System (Roche/Hitachi). Briefly, total bilirubin, in the presence of a suitable solubilizing agent, is coupled with a diazonium ion in a strongly acidic medium to form red-colored azobilirubin. The color intensity of the formed dye is proportional to the concentration of TB present in the sample. It was determined photometrically by the increase in absorbance at 552 nm.

### SNP genotyping and quality control

We performed genome-wide genotyping on 964 GOCS cohort participants and 8 duplicate genotyping controls from the Human Genome Diversity Cell Line Panel (HGDP-CEPH) using the Infinium^®^ Multhi-Ethnic Global BeadChip (Illumina. Inc). We loaded genotyping data into GenomeStudio v2.0.3 (Illumina. Inc) and called automatic clustering (Tobar et al., 2019; Miranda et al., 2022). From the total of samples analyzed, 14 with a call rate <0.98 were removed. The average genotyping concordance between the HGDP-CEPH Panel duplicate controls was 99.99% ± 0.02, and the total genotyping rate was 0.9993. The project was exported to PLINK format for further filtering.

Using PLINK v1.9 (Purcell et al., 2007), we removed three more samples based on gender mismatch and three due to relatedness (IBD/IBS>0.2). Excluded variants had minor allele frequency (MAF) < 0.01, missing genotype data >5%, duplicated physical positions (one variant was kept from each duplicate pair), and deviations from the Hardy-Weinberg Equilibrium (HWE) (*p* < 1 × 10^−4^). To rule out strand inconsistency, 58,236 transversions (A↔T or C↔G) were removed. After these filters, we obtained 944 participants (468 males and 476 females) and a dataset of 700,344 autosomal variants in the GRCh37 human reference sequence.

### Genotype imputation

We used the filtered data set of 700,344 variants for genotype imputation in the TOPMed Imputation Server (Das et al., 2016; Taliun et al., 2021). The TOPMed imputation pipeline used Minimac4 v1.6.6 (Fuchsberger et al., 2015), including Eagle v2.4 for phasing genotypes, the TOPMed r2 reference panel with 97,256 samples from diverse populations, and more than 300 million genetic variants. We used BCFTools v1.9 to obtain imputed single nucleotide variants with high imputation probability (rsq>0.9) and MAF >0.005, yielding a total of 7,797,326 variants in GRCh38 human reference sequence.

### Construction of the multiethnic panel for local ancestry

We obtained the genotypes from the Human Genome Diversity Project (HGDP) (Bergström et al., 2020) and the 1000 Genomes Project (1KGP) (Auton et al., 2015), contained in the gnomAD v3.1.2 release (https://gnomad.broadinstitute.org/downloads). The data set included 4,150 samples and about 150 million single nucleotide variants. Using BCFTools, we filtered this data set to keep only passing filter variants with genotype called in at least 90% of the samples and with a frequency greater than 0.5%, obtaining 15,520,451 variants. Using BCFTools, we intersected by equal position and alleles this dataset with the imputed dataset for the 944 GOCS participants.

In the resulting dataset containing 5,094 samples, we used PLINK v1.9 to remove regions in high linkage disequilibrium (Price et al., 2006). Data were pruned using an independent pairwise approach with a window size of 50 kb, a step size of 5 SNPs, and a r2 cutoff threshold of 0.2. With the 254,807 variants obtained, we estimated global ancestry using ADMIXTURE with cross-validation and several populations *K* from 3 to 18 (Alexander et al., 2009). Global ancestry composition was plotted in RStudio v2022.07.0. Using PLINK v1.9 on the same variants, we estimated 20 principal components (PCs) for population stratification correction.

For the local ancestry panel, we selected 676 samples with an estimated ancestry component with an origin of >97% for Native American, >97% for European, and >99.9% for African (Supplementary Tables S2, S3).

To obtain phased genotypes and ensure that we had harmonized genotypes between the different data sets, the set with the 5,094 samples was imputed again on the TOPMed imputation server. From these data, we prepared a subset with the 944 GOCS samples for local ancestry (imputation probability >99% and MAF>0.05%). We performed a second subset with the 676 reference panel samples and selected the variants described in gnomAD to estimate local ancestry in the Latino admixed population.

### Local ancestry estimation and local ancestry deconvolution in GOCS

To estimate local ancestry in GOCS, we used RFMix v2 (Maples et al., 2013) with our reference panel of 676 samples with the highest Native American, European, or African global ancestry. To estimate global ancestry from local ancestry estimates, we performed a weighted sum by the size of each autosome. We used Tractor (Atkinson et al., 2021) to deconvolute GOCS genotypes in the different ancestry. The tractor pipeline takes the RFMix results, extracts each ancestry’s inferred haplotypes (or tracts), and generates an ancestry-isolated VCF files.

### Admixed ancestry and local ancestry deconvoluted GWAS (LAD-GWAS)

We developed a traditional admixed ancestry GWAS with the 707 GOCS participants using PLINK2 in association with serum TB. We considered 3,744,716 variants with at least 5 participants with alternative homozygous genotypes to fit additive models.

To assess the effect of the isolated Native American (NAT) or European ancestry (EUR) in GOCS, we used PLINK2, considering half-calls as missing. We only consider 2,604,077 variants for NAT ancestry and 3,229,387 variants for EUR ancestry. These variants had at least 3 participants with alternative homozygous genotypes to fit an additive model.

Inferred variants of AFR origin were excluded from the analysis due to the low proportion found in GOCS. Non-autosomal variants were excluded. Manhattan and Q-Q plots were constructed using the “qqman” library on RStudio.

To assess whether our local ancestry estimates were correct, we compared the allele frequencies of the top variants among the entire GOCS cohort and the estimated NAT or EUR ancestry with the frequencies obtained in gnomAD v3.1.2 genomes. We selected frequencies for admixed Latino ancestry (AMR), Europeans (non-Finnish), and Native American ancestry estimated with a similar methodology (https://gnomad.broadinstitute.org/). We also compared the allele frequencies of our variants with those described in the HGDP project for NAT ancestry, which includes samples of Maya, Colombian, Karitiana, Pima, and Surui origin.

### Statistical methods

We calculated summary statistics in the GOCS cohort using RStudio. We used a two-tailed Student’s t-test to compare boys and girls. The admixed ancestry GWAS was conducted with 707 GOCS participants using an additive genetic model adjusted by sex, age, BAZ, and five genetic principal components (PCs). In contrast, for the LAD-GWAS, we adjusted by sex, age, and BAZ as covariates. Associations with *p* < 5 × 10^−8^ were considered genome-wide significant, while associations between *p* < 1 × 10^−5^ and >5 × 10^−8^ were considered suggestive of association. Only variants corrected for multiple testing with FDR-BH *p* < 0.05 are reported. We use Pearson´s correlation to compare allele frequencies between the different ancestries. All analyses were performed on an HP Z800 Workstation, with 24x Intel^®^ Xeon(R) CPU X5675 @ 3.07 GHz, 48 Gb RAM, and a 6 Tb RAID, with Ubuntu 20.04.4 LTS 64-bit operating system.

## Results

### Total serum bilirubin levels among participants of the study

The 707 participants of the Chilean GOCS cohort included in the present study had an average age of 15.4 ± 0.98 years, and 51.8% were females (Table 1). Total bilirubin levels were significantly higher in boys than girls. On the other hand, BAZ was considerably higher in girls than boys. To manage confounding, the additive regression models of the GWAS were adjusted for age, sex, and BAZ.

**TABLE 1:** Characteristics of the 707 GOCS Cohort participants included in this study. Significant differences were found for age and BAZ according to sex. These covariates were included in the fit of the GWAS regression models.

	Boys	Girls	*p*-value*
N (%)	341 (48.2%)	366 (51.8%)	
Age (years)	14.93 ± 0.91	15.77 ± 0.86	**4.93x10** ^**−33**^
BMI for age z-score (BAZ)	0.63 ± 1.21	0.89 ± 1.08	**1.92x10** ^**−3**^
Total bilirubin (mg/dL)	0.61 ± 0.44	0.39 ± 0.29	**4.97x10** ^**−15**^

*In bold, significant differences between boys and girls; Student’s t-test.

### Global ancestry composition of GOCS participants

Using the ADMIXTURE program with cross-validation, we estimated that the optimal number of populations for the 5,094-sample dataset, including participants from GOCS, the HGDP, and the 1KGP, is obtained with K = 15 (Supplementary Figure S1).

**FIGURE 2:**
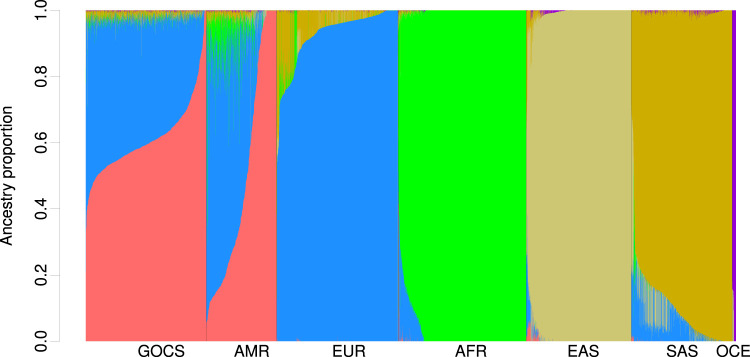
Global ancestry proportions are estimated in GOCS, the HGDP, and 1KGP. Global ancestry was estimated with ADMIXTURE using K = 15 populations and included probably Chilean Mapuche, Peruvian, Mexican, Colombian, Surui, Maya, Karitiana, and Pima ( ◆ RED); Iberian, Toscani, Basque, Sardinian, Finnish, British, Northern European, Russian, Orcadian, Bedouin, Druze, Mozabite, and Palestinian ( ◆ BLUE); Gambian, Mende, Esan, Yoruba, Luhya, African American in Southwest US, African Caribbean in Barbados, Bantu, Pygmy, and San ( ◆ GREEN); Japanese, Hezhen, Daur, Oroqen, Yakut, Xibo, Mongola, Yi, Tu, Naxi, Uygur, Dai, Kin, Lahu, Han, Cambodian, She, Miao, and Tujia ( ◆ LIGHT BROWN); Indian, Sri Lankan, Punjabi, Bengali, Guajarati, Burusho, Pathan, Sindhi, Kalash, Brahui, Balochi, Makrani, and Hazara ( ◆ DARK BROWN); Melanesian, and Papuan ( ◆ PURPLE). Regions included admixed Latino (AMR), Europeans (EUR), Africans (AFR), East Asians (EAS), Central/South Asians (SAS), and Oceanian (OCE).

The GOCS cohort showed a global Native American (NAT) ethnic component that reached 61.4% (Supplementary Table S1; Figure 2). This regional component was separated into three different populations, with a main contribution that could come from the Native-American Mapuche population (55.3%) and a low contribution from Andean (3.84%) and Central American origin (2.2%) (Supplementary Table S1; Supplementary Figure S2).

The global European (EUR) ancestry component averaged a total of 36%, coming mainly from Iberian, Basque, Sardinian, Tuscan, Italian, and French populations (25%) and, to a lesser extent, from Northern Europeans (6.2%) (Supplementary Table S1). Because we included HGDP samples of Middle Eastern origin, we could disentangle this component and estimate that it is present at an average of 4.75% in GOCS. As expected, the GOCS cohort had a low ancestry component of AFR origin (0.55%), East Asian (0.34%), Central and South Asian (0.16%), and Oceanian (0.13%) (Supplementary Table S1).

### Local ancestry in GOCS

Based on the global ancestry estimate of the 5,094 samples, we selected 676 samples with the highest proportion of NAT, EUR, or AFR ancestry (Supplementary Tables S2, S3). Among them, 16 samples in GOCS showed a high component of NAT origin. Additionally, we selected samples from the HGDP and the 1KGP, reaching a total of 111 samples with a percentage of NAT ancestry greater than 97%. The average NAT component in these 111 samples reached 99.7% (Supplementary Table S3).

For the EUR component, we selected 326 samples with more than 97% European global ancestry, including the HGDP and the 1KGP samples from Basque, Bedouin, Northern Europeans, Finnish, French, British, Iberia, Arcadian, and Sardinian populations. The average EUR ethnic component in these samples reached 98.7%.

Additionally, 239 samples for our reference panel with AFR origin averaged 99.9% global ancestry in the HGDP and the 1KGP. These samples belong to Esan, Gambian, Pygmy, and Yoruba populations.

According to the local ancestry results obtained with RFMix, GOCS participants are composed of 45.6% NAT ancestry, 52.5% EUR ancestry, and 1.9% AFR ancestry (Supplementary Table S4). Although differences are observed for the estimated global ancestry with ADMIXTURE, both results are highly agreed (*R* ^2^ = 0.998 for NAT, *R* ^2^ = 0.985 for EUR, and *R* ^2^ = 0.661 for AFR ancestry, respectively). In general, ADMIXTURE could overestimate the global NAT component and underestimate the EUR and AFR components in GOCS, considering that the participants of the GOCS cohort have a recently admixed ancestry component and we did not have available samples of pure NAT ancestry to include in the panel for the estimation of global ancestry. (Supplementary Figure S4).

### GWAS of total bilirubin in GOCS participants

**FIGURE 3:**
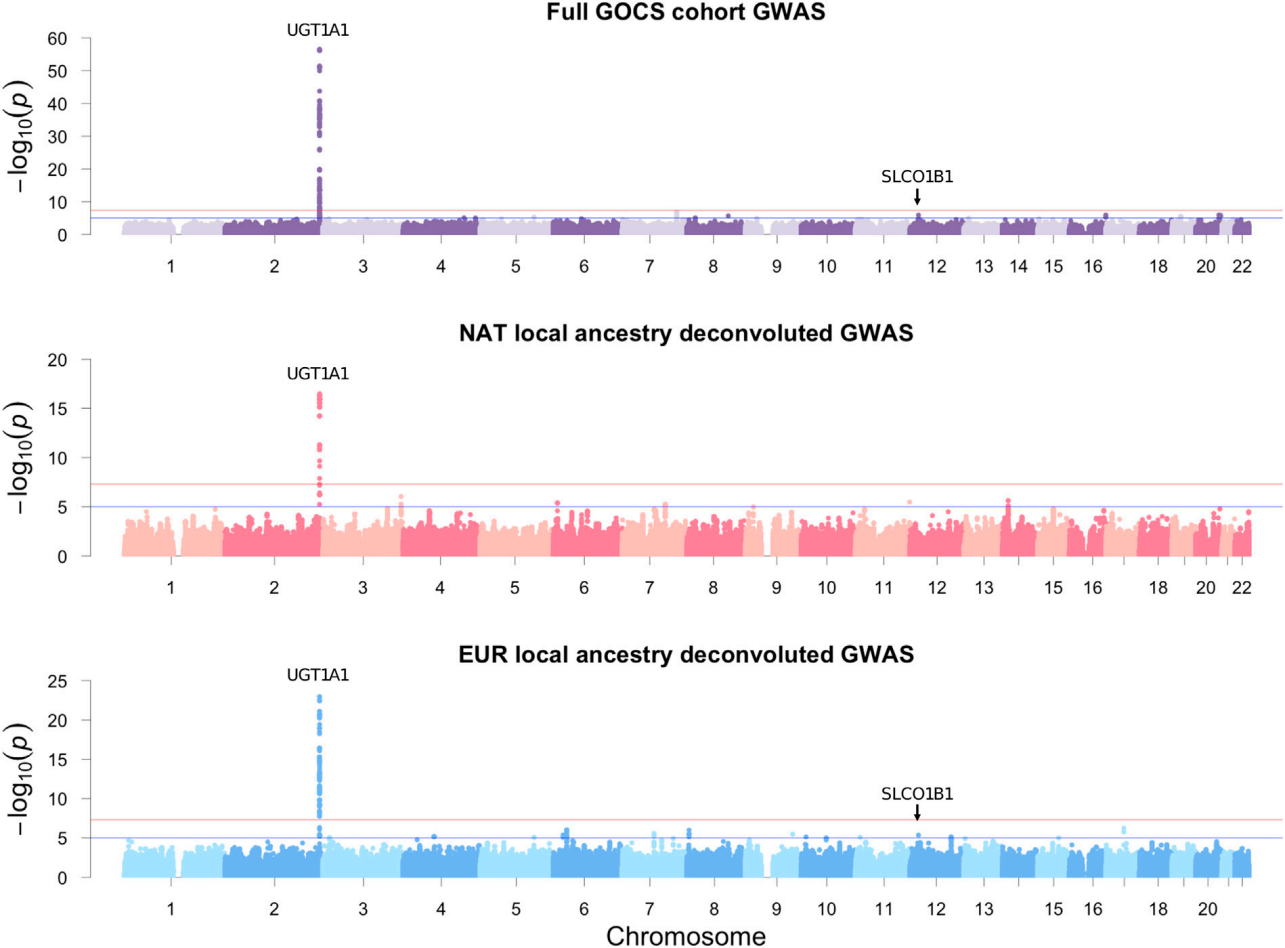
GOCS cohort GWAS and LAD-GWAS of total bilirubin. Additive models were adjusted for age, sex, and BAZ. The traditional GWAS of the GOCS cohort was also adjusted for five genetic principal components. Only autosomal variants were included.

**FIGURE 4:**
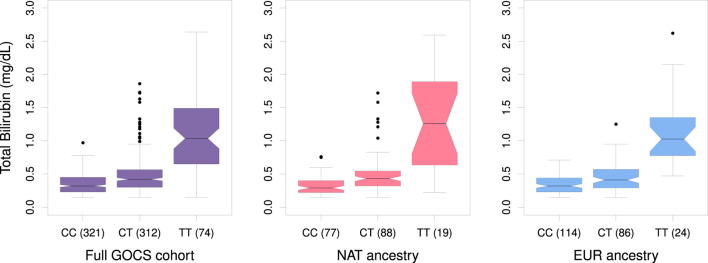
Boxplot of total bilirubin levels in GOCS according to genotypes of the variant rs887829 near UGT1A1 for the total cohort and for sub-cohorts with Native American or European ancestry. In parentheses, the number of participants carriers of each genotype is shown.

Gene variants reaching the threshold of significance at the genome-wide level (*p* < 5 × 10^−8^) were limited to the vicinity of the UDP-glucuronosyltransferase gene family 1 member A1 (*UGT1A1*) (Figure 3; upper). The strongest association was with the rs887829 variant upstream *UGT1A1*, beta = 0.3 mg/dL total bilirubin, *p* = 3.34 × 10^−57^, effect allele T (Table 2). This variant masked other 232 significant variants in high linkage disequilibrium (*R* ^2^ > 0.1) located in the vicinity (±500 Kb), and its allele frequency in GOCS was 32.5% (Supplementary Table S5). Carriers of the TT genotype of the rs887829 variant averaged 3.32 times higher bilirubinemia than those of the CC genotype (*p* = 2.9 × 10^−34^) (Figure 4).

**TABLE 2:** Summary statistics of the GWAS for the entire GOCS cohort and the Local Ancestry Deconvoluted GWAS for total bilirubin levels.

Chr	Position	Variant	Candidate genes	Ref	Alt	Beta (mg/dL)	95% CI	*p-value*	N	Masked variants
*Full GOCS Cohort GWAS*										
2	233620476	rs1551285	*UGT1A1*	A	C*	0.14	0.10; 0.18	3.45 × 10^−10^	707	20
2	233640580	rs28969691	*UGT1A1*	T	C	0.26	0.20; 0.31	5.58 × 10^−17^	707	33
2	233759924	rs887829	*UGT1A1*	C	T	0.30	0.26; 0.33	3.34 × 10^−57^	707	232
2	233794259	rs10169532	*UGT1A1*	C	T	0.09	0.05; 0.13	2.55 × 10^−6^	707	1
2	233823665	rs17868361	*UGT1A1*	G	A	0.13	0.07; 0.18	3.13 × 10^−6^	707	24
4	150375690	rs116206753	*LRBA*	G	A	0.18	0.10; 0.26	8.75 × 10^−6^	707	30
4	178687395	rs76776133	-	T	A	0.08	0.05; 0.12	9.62 × 10^−6^	707	218
5	133713935	rs10900821	*FSTL4*	C	T	0.15	0.09; 0.22	6.04 × 10^−6^	707	55
7	134409273	rs1646747	*AKR1B1*	G	A	0.13	0.08; 0.18	1.40 × 10^−7^	707	177
8	21091713	rs34437032	*GFRA2*	G	A	0.10	0.05; 0.14	9.27 × 10^−6^	707	105
8	102540688	rs112547984	*ODF1*	G	A	0.17	0.10; 0.24	2.21 × 10^−6^	707	78
12	21053900	rs1910167	SLCO1B1**	T	C	0.16	0.10; 0.23	1.26 × 10^−6^	707	368
16	89583267	rs455868	*CPNE7*	G	A*	−0.11	−0.15; −0.06	1.18 × 10^−6^	707	85
19	21701468	rs34426376	*ZNF100*	C	T	0.14	0.08; 0.20	2.92 × 10^−6^	707	36
20	57417868	rs4810066	*RBM38*	T	C*	0.11	0.07; 0.16	1.11 × 10^−6^	707	23
20	60990081	rs76031163	*CDH4*	C	G	0.13	0.08; 0.19	1.35 × 10^−6^	707	51
***NAT local ancestry deconvoluted GWAS***										
2	233759924	rs887829	*UGT1A1*	C	T	0.35	0.28; 0.43	3.29 × 10^−17^	184	202
3	193041235	rs9813423	*MB21D2*	G	A	0.20	0.13; 0.28	9.11 × 10^−7^	210	54
6	10230583	rs79896759	*OFCC1*	A	G	0.22	0.13; 0.30	4.12 × 10^−6^	186	33
7	106045833	rs6962785	*CDHR3*	T	C*	−0.23	−0.33; −0.14	5.48 × 10^−6^	166	81
9	19759310	rs1359822	*SLC24A2*	T	C*	−0.22	−0.32; −0.13	1.05 × 10^−5^	193	46
11	133984164	rs536866	*IGSF9B*	A	G	0.19	0.11; 0.27	3.30 × 10^−6^	168	103
14	33690956	rs4627235	*NPAS3*	G	A	0.19	0.11; 0.26	2.39 × 10^−6^	188	140
***EUR local ancestry deconvoluted GWAS***										
2	233759924	rs887829	*UGT1A1*	C	T	0.28	0.23; 0.32	1.14 × 10^−23^	224	228
3	16227479	rs14576	*GALNT15*	C	A	0.15	0.09; 0.21	9.05 × 10^−6^	211	104
4	76456075	rs13147930	*SHROOM3*	C	T	0.23	0.13; 0.32	6.63 × 10^−6^	200	273
5	133713935	rs10900821	*FSTL4*	C	T	0.24	0.14; 0.34	8.28 × 10^−6^	214	82
6	24075096	rs1592334	*NRSN1*	G	A	0.22	0.13; 0.31	4.14 × 10^−6^	224	55
6	33131559	rs41288897	*COL11A2*	T	A	0.19	0.12; 0.26	1.03 × 10^−6^	239	216
7	78221035	rs1107560	*MAGI2*	G	T	0.31	0.18; 0.43	2.69 × 10^−6^	220	42
8	5721494	rs71523681	*CSMD1 MCPH1*	T	G	0.23	0.14; 0.31	1.00 × 10^−6^	233	15
9	116777560	rs4837851	*ASTN2*	T	C*	−0.27	−0.38; −0.16	3.24 × 10^−6^	211	8
10	11684554	rs12414558	*ECHDC3*	G	A	0.22	0.12; 0.31	7.33 × 10^−6^	202	25
10	61584791	rs2606106	*CABCOCO1*	A	C	−0.16	−0.23; −0.09	9.94 × 10^−6^	210	165
11	12027127	rs4391796	*DKK3*	T	C	0.18	0.10; 0.25	8.21 × 10^−6^	214	50
12	21053900	rs1910167	SLCO1B1**	T	C	0.24	0.14; 0.34	4.35 × 10^−6^	215	431
12	101694159	rs2695289	*MYBPC1*	G	T*	−0.18	−0.25; −0.10	7.39 × 10^−6^	228	310
15	75081804	rs12901092	*PPCDC*	C	A	−0.15	−0.22; −0.09	9.68 × 10^−6^	204	266
17	44192700	rs76335095	*ATXN7L3*	G	T	0.33	0.20; 0.45	5.61 × 10^−7^	206	2

**Ref/Alt**: reference and alternative alleles, respectively. Alternative alleles are tested alleles, except for variants marked with *. **N**: number of participants included in the GWAS., Masked variants are significant but in high linkage-disequilibrium (*R* ^2^ > 0.1) and up to 500 kb to the index variant. **Corresponds to candidate genes *SLCO1B1, SLCO1B3*, and *SLCO1B7*. All the selected variants are FDR-BH, significant (*p* < 0.05). Genomic positions are according to the GRCh38 Human Genome Assembly.

In addition to *UGT1A1*, we found 11 other regions with a suggestive association (*p-value* between 1 × 10^−5^ and 5 × 10^−8^). Among the suggestive variants, we found the rs1910167 variant, effect allele C, in the solute carrier organic anion transporter family member 1B3 (*SLCO1B3*), 1B7 (*SLCO1B7*), and upstream of 1B1 (*SLCO1B1*), beta = 0.16 mg/dL total bilirubin, *p* = 1.26 × 10^−6^. This variant masked 368 other significant variants in the vicinity in high linkage disequilibrium and has a frequency of 8.3% in GOCS (Supplementary Table S4).

The variant rs887829 near *UGT1A1* explained 34.98% of the variation in total bilirubin levels, while the rs1910167 variant near *SLCO1B1* only explained 4.61%. When considering the combined effect, both variants explained 36.86% of the variation in bilirubin levels in the entire cohort.

Other variants suggestive of association were found in candidate genes *LRBA*, *FSTL4*, *AKR1B1*, *GFRA2*, *ODF1*, *CPNE7*, *ZNF100*, *RBM38*, and *CDH4* (Table 2). The GWAS genomic inflation factor was λ = 1.008 (Supplementary Figure S4).

### Local ancestry deconvoluted GWAS of total bilirubin in GOCS participants

By considering the deconvoluted genotypes with NAT origin, we found that the variant with the strongest association was also rs887829, upstream of the *UGT1A1* gene, beta = 0.35 mg/dL total bilirubin, *p* = 3.29 × 10^−17^, with a frequency of 34.2% in the NAT component of GOCS (Table 2; Supplementary Table S5). We also found six other regions with variants suggestive of association near *MB21D2*, *OFCC1*, *CDHR3*, *SLC24A2*, *IGSF9B*, and *NPAS3* genes (Table 2). When we performed the same analysis but considering the inferred haplotypes for EUR ancestry, the variant with the strongest association was also rs887829, upstream of *UGT1A1*, beta = 0.28, *p* = 1.14 × 10^−23^, with a frequency of 32.1% in the EUR component of GOCS (Table 2; Supplementary Table S5).

We found 15 other regions with variants suggestive of association. One of these regions included the rs1910167 variant in *SLCO1B3/SLCO1B7* and upstream of *SLCO1B1*, beta = 0.24 mg/dL total bilirubin, *p* = 4.35 × 10^−6^. This variant has an allele frequency of 14.2% in the EUR component of GOCS but only 0.3% in the NAT component (Table 2; Supplementary Table S5). Other variants suggestive of association were in the candidate genes *GALNT15*, *SHROOM3*, *FSTL4*, *NRSN1*, *COL11A2*, *MAGI2*, *CSMD1/MCPH1*, *ASTN2*, *ECHDC3*, *CABCOCO1*, *DKK3*, *MYBPC1*, *PPCDC*, and *ATXN7L3* (Table 2).

The genomic inflation factor was λ = 1.01646 for NAT ancestry and λ = 1.01646 for EUR LAD-GWAS (Supplementary Figure S4).

In general, carriers of the TT genotype of rs887829 in the NAT ancestry of GOCS averaged 4-fold higher bilirubinemia than carriers of the CC genotype (*p* = 2.82 × 10^−12^). In contrast, carriers of the TT genotype in the EUR ancestral component of GOCS averaged 3.2 times higher bilirubinemia (*p* = 1.09 × 10^−27^) (Figure 4).

For the GWAS with the NAT ancestral component of GOCS, the rs887829 variant near *UGT1A1* explained 37.6% of the variation in bilirubin levels. When considering the GWAS with the EUR ancestry of GOCS, this variant explained 28.4% of the variation. In comparison, the rs1910167 near *SLCO1B1* variant explained 12.3%, and the combination of rs887829/rs1910167 explained 42% of the variation in bilirubin levels.

When we compared the allele frequencies of the top variants between the entire GOCS cohort and the estimated NAT or EUR ancestry, we found relevant differences. The variant rs28969691, close to *UGT1A1*, was highly prevalent in NAT ancestry (19.8%) but almost absent in EUR (0.9%). Conversely, variants close to *SLCO1B1*, *FSTL4*, *RBM38*, and *CSMD1* genes are virtually absent in NAT ancestry (Supplementary Table S5).

The frequency of the top variants in the GOCS cohort correlated very well with those described in gnomAD for admixed Latin ancestry (Pearson´s r = 0.99). Still, the correlation was lower when compared with the NAT or EUR ancestry estimated in GOCS (Pearson´s r = 0.93 and r = 0.95, respectively). The frequencies of NAT variants estimated in GOCS were similar to those estimated in gnomAD (Pearson´s r = 0.96) and those in the HGDP (Pearson´s r = 0.97). For the estimated EUR ancestry in GOCS, there was a high correlation with the frequency of variants in gnomAD for non-Finnish Europeans (Pearson´s r = 0.99) (Supplementary Table S5).

## Discussion

Although association studies to describe the genetic determinants of bilirubin levels in different populations began more than 10 years ago, the effect and magnitude of such genetic variants in Native American ancestry was unknown. The admixed Latino population has NAT, EUR, and AFR ancestry components, so building cohorts with pure NAT ancestry can be highly complex and costly. In the present study, we used Local Ancestry Deconvolution to isolate chromosomal fragments of NAT origin of Chilean adolescents and performed a GWAS with serum bilirubin levels. Our results showed for the first time that *UGT1A1* is also the most strongly associated gene with serum bilirubin levels in NAT ancestry, suggesting several differences in the magnitude of association compared to EUR ancestry.

### Global and local ancestry in GOCS

As a result of the global ancestry analysis, we found that NAT ancestry was separated into three subpopulations. One subpopulation grouped samples with Central American origin; a second subpopulation grouped the 1KGP participants of Peruvian origin, which we called the Andean subpopulation, while a third subcomponent we believe corresponds to Mapuche origin. We make this statement since most GOCS participants cluster in this group, and we previously determined that the main component of NAT origin in our cohort is of Mapuche origin and marginally of Aymara origin (Vicuña et al., 2021).

Considering this, constructing a reference panel for local ancestry based only on genotypes obtained from the 1KGP and HGDP projects would greatly bias our results because the ancestry component of Mapuche origin is practically non-existent in those participants (Supplementary Figure S2). Therefore, we decided to include 16 GOCS participants with a higher proportion of NAT ancestry in our panel to account for haplotypes of Mapuche origin.

Although it was initially described that the origin of Chileans is predominantly NAT, EUR, and to a lesser extent, AFR (52%, 45%, and 3%, respectively) (Eyheramendy et al., 2015), recent studies suggest that there is also a southern Mediterranean or Middle East ancestry component, as a product of the migration of Christian converts of non-European origin that dates to the Spanish colonization of the Americas (Chacón-Duque et al., 2018). This study mentions that Sephardic/East/South Mediterranean ancestry averages 4% in the Chilean population (Chacón-Duque et al., 2018). By including the HGDP samples in our global ancestry estimate, which contains 162 participants of Middle Eastern origin (Bedouin, Druze, Mozabite, and Palestinian), we could dissect this component in the GOCS cohort. It averaged 4.75%, which is quite close to that reported by Chacón-Duque et al. (Supplementary Table S1). For simplicity of analysis, we considered Middle East ancestry as part of the Europeans in this work.

We found a high degree of agreement when we compared the global ancestry with the local ancestry estimates. However, ADMIXTURE could be overestimating the proportion of NAT ancestry. This will occur because of the lack of pure Mapuche or Aymara ancestry samples to include in our reference panel for a better definition of the outgroup. When considering the 16 GOCS samples used in our reference panel, the proportion of NAT ancestry estimated by RFMix ranged from 75% to 96%, with an average of 82.9% (Supplementary Table S3). Despite the differences obtained between ADMIXTURE and RFMix, our estimates are valid considering the high agreement for NAT and EUR ancestry (*R* ^2^ = 0.99 for each, Supplementary Figure S3).

#### Traditional GWAS and LAD-GWAS of serum bilirubin

Although the standard practice is to use local ancestry as an adjustment variable in regression models to reduce the confounding effect of population stratification by ethnicity, our focus of analysis is different. Because our goal was to determine the effect of NAT ancestry on the regulation of bilirubin levels, rather than adjusting, we stratified the GOCS cohort into its different ancestral components.

When we used the Tractor pipeline to extract the haplotype phases and separate them according to NAT, EUR, or AFR origin, several fragments (or tracts) with discordant ancestry (or half-call) were generated. Although we could have treated this discordant ancestry as haploid for GWAS, we decided to approach the analysis more conservatively and treat them as missing due to our reduced sample size. In practice, with the Tractor pipeline, we generated a sub-cohort for each variant, and the participants of these sub-cohorts are all those who called a diploid genotype for that variant in a specific ancestry; therefore, genotypes for an individual are represented in a (3 x *n*)_ijk_ matrix, where 3 is the possible allele combinations (reference/reference, reference/alternative, and alternative/alternative), and *n* is the number of variants that called for a diploid genotype in ancestries *i*, *j*, or *k*. Therefore, the number of participants analyzed in each LAD-GWAS was variable for each variant, ranging between 166 and 239 for the variants in Table 2 with estimated origin in NAT or EUR ancestry, unlike the 707 participants included in the traditional GWAS.

Both in the entire cohort GWAS and the LAD-GWAS, we found that the most significantly associated variant was rs887829. This variant is in the promoter of the *UGT1A1* gene, 310 bp upstream of the (TA)_n_ repeat UGT1A1*28 (rs3064744) at the TATA box, which is known to reduce the expression of UGT1A1 enzyme on Gilbert-Meulengracht´s Syndrome, drifting into reduced bilirubin glucuronidation (Bosma et al., 1995; Shin et al., 2015). Glucuronidation is an essential mechanism in forming water-soluble substrates not limited to bilirubin but many xenobiotics, leading them to excretion from the body via bile or urine (Tephly and Burchell, 1990). Thus, *UGT1A1* as well as other UGT genes have important roles in drug/xenobiotic and polyphenol metabolism (An et al., 2015; Gammal et al., 2016; Allegra et al., 2017; Xu et al., 2017; Leger et al., 2018). Recent research has shown that high bilirubin levels within normal ranges are strongly associated with a lower prevalence of diseases mediated by oxidative stress, including diabetes, metabolic syndrome, and cardiovascular disease (Lin et al., 2009b; Jylhävä et al., 2012; Cox et al., 2013; Benton et al., 2015).

Among the variants suggestive of association, it is important to mention those in *SLCO1B3/SLCO1B7* genes and upstream *SLCO1B1*, both for the entire GOCS participants and for the LAD-GWAS with EUR ancestry, but not for the LAD-GWAS with NAT ancestry. *SLCO1B1* is a hepatic transporter with an affinity for bilirubin and is associated with the levels of this molecule in multiple studies with participants of European and Middle East ancestry (Johnson et al., 2009; Sakaue et al., 2021; Sinnott-Armstrong et al., 2021).

Even when we found variants suggestive of association in other genes, none of them have been described in other populations, both for the traditional GWAS and for the LAD-GWAS, without *a priori* biological plausibility for these results.

The high correlation between variants of NAT estimated in GOCS with those estimated for Amerindigenous in gnomAD and those of NAT in the HGDP project partially validate the deconvolution of local ancestry performed in our study. However, a wider analysis is required to perform a comprehensive estimate of variant deconvolution.

When we compared the frequency of variants between NAT and European ancestry, we found important differences. This observation suggests that polygenic risk scores or the instrumental variables in Mendelian Randomization studies, might not have direct applicability when used in populations genetically different from those for which they were described. A local ancestry deconvolution method is an interesting approach that allows identifying variants with significantly different frequencies between populations to adjust these genetic instruments and achieve greater applicability.

### Strengths and weaknesses of this study

The present study is the first to report the isolated effect of NAT ancestry in the regulation of serum bilirubin levels, and the results are consistent with those described in other regions, such as Europe, Africa, Asia, and Oceania. One of the weaknesses of the study is the relatively small sample size, which prevents obtaining information on variants such as those described in the *SLCO1B1, SLCO1B3, and SLCO1B7* genes, significant in the European population, but with shallow allelic frequency in the NAT component. Another drawback is that we cannot confirm whether new players, different from *UGT1A1,* are relevant in NAT ancestry for bilirubin regulation due to the lack of a cohort to replicate our suggestive findings.

## Conclusion

We show that the local ancestry deconvolution method allows to efficiently isolate a component of ancestry in admixed populations. Using this approximation, we were able to determine that variants in the *UGT1A1* gene are the most strongly associated with serum bilirubin levels in the inferred Native American ancestry. Our results confirm the pan-ethnic relevance of *UGT1A1* in regulating bilirubin levels and illustrates the general value of the local ancestry deconvolution approach to assess isolated ancestry effects in admixed populations.

## Data Availability

The data presented in the study are deposited in the UC Research Data Repository (https://doi.org/10.60525/04teye511/JLG0FV).
