# Supplementary material for: Genetic determinants of serum bilirubin using inferred native American gene variants in Chilean adolescents
Source: Front Genet. 2024 May 17;15:1382103. doi: 10.3389/fgene.2024.1382103 (PMC11140026; doi:10.3389/fgene.2024.1382103)
Supplement: Supplementary file 1 [file DataSheet1.docx]

SUPPLEMENTARY MATERIAL

**Title: Genetic determinants of serum bilirubin using inferred Native American variants derived from local ancestry deconvolution.**

José P. Miranda^1,2,3^, Ana Pereira^4^, Camila Corvalán^4^, Juan F. Miquel^5^, Gigliola Alberti^6,7^, Juan C. Gana^6,7^, and José L. Santos^1,2*^.

^1^Department of Nutrition, Diabetes, and Metabolism, School of Medicine, Pontificia Universidad Católica de Chile, Santiago 8331150, Chile.

^2^School of Medicine, PhD in Epidemiology Program, Pontificia Universidad Católica de Chile, Santiago 8331150, Chile.

^3^*Advanced Center for Chronic Diseases (ACCDiS), Pontificia Universidad Católica de Chile & Universidad de Chile, Santiago 8331150, Chile.*

^4^*Instituto de Nutrición y Tecnología de los Alimentos INTA, Universidad de Chile, Macul 7830490, Chile.*

^5^*Department of Gastroenterology, School of Medicine, Pontificia Universidad Católica de Chile, Santiago 8331150, Chile.*

^6^*Pediatrics Division, School of Medicine, Pontificia Universidad Católica de Chile, Santiago 8331150, Chile.*

^7^*Department of Gastroenterology and Pediatric Nutrition, School of Medicine, Pontificia Universidad Católica de Chile, Santiago 8331150, Chile.*

***Correspondence**

**José L. Santos;** [**jsantosm@uc.cl**](mailto:jsantosm@uc.cl)

**Supplementary Figure 1**. **Cross-validation error at different numbers of K populations**. Using ADMIXTURE, global ancestry was estimated across GOCS participants, including participants from the 1KGP and HGDP projects. Cross-validation error was lower at K=15.

**Supplementary Figure 2.** **Ancestry proportions are estimated in GOCS, 1KGP, and HGDP**. Detail on Native American ancestry, estimated with ADMIXTURE using K=15. Populations included probably Chilean Mapuche (♦); Andean Peruvian (♦); Centro American Mexican, Colombian, Surui, Maya, Karitiana, and Pima (♦); and other ancestries (♦).

**Supplementary Figure 3. Comparison between global and local ancestry estimations in GOCS for Native American (NAT), European (EUR), and African (AFR) ancestry**. R^2^=0.99 for NAT, R^2^=0.99 for EUR, and R^2^=0.66 for AFR, respectively.

**Supplementary Figure 4**. **Q-Q plot of GWAS *p values***. Genomic inflation factor in the entire GOCS cohort GWAS, and the Native American (NAT) or European (EUR) local ancestry deconvoluted subcohorts was λ=1.008, λ=1.01646, and λ=1.00052, respectively.


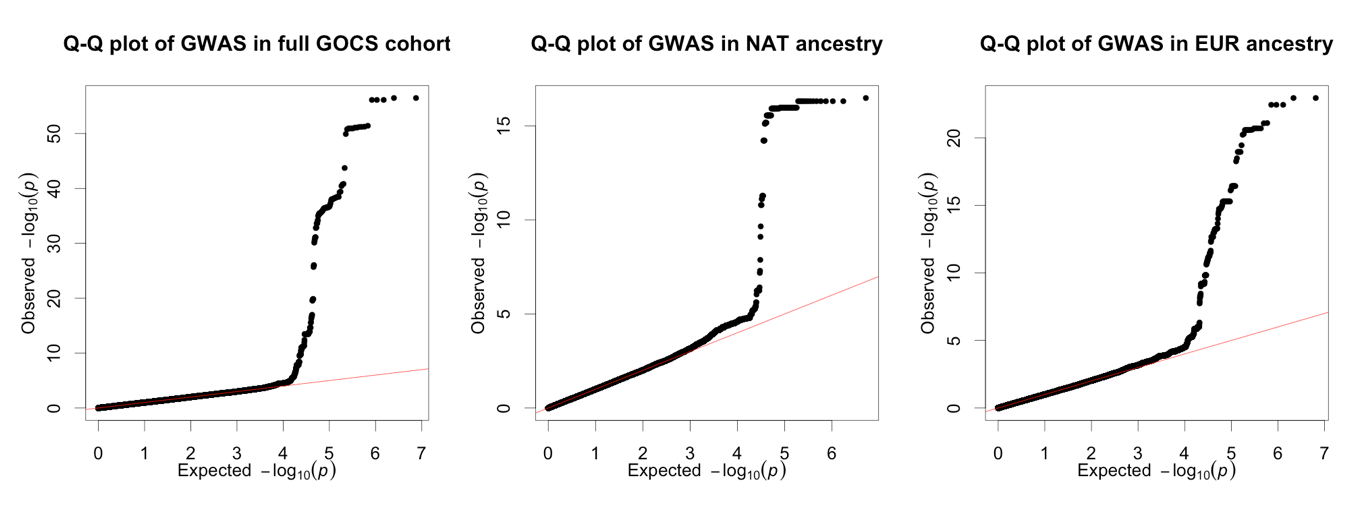


**Supplementary Table 1. The average proportion of global ancestry for the 944 GOCS participants.** Proportions are presented at the Region, and Population-level estimated with K=15 using ADMIXTURE.

| **Region** | **Population** | **Estimated Ancestry Proportions** |
| --- | --- | --- |
|  | Probably Mapuche | 0.5529 |
| Americas | Peruvian | 0.0384 |
|  | Colombian, Surui, Maya, Karitiana, Pima, Mexican, Puerto Ricans | 0.0222 |
|  | **Total American** | **0.6135** |
|  | Iberian, Basque, Sardinian, Toscani, French, Italian | 0.2503 |
| Europe | Finnish, British, Russian, Orcadian, Northern and Western Europe | 0.0624 |
|  | Bedouin, Druze, Mozabite, Palestinian | 0.0475 |
|  | **Total European** | **0.3603** |
|  | Pygmy, San, Bantu, Mende | 0.0005 |
| Africa | Esan, Yoruba, African Caribbean | 0.0017 |
|  | Gambian Mandinka | 0.0016 |
|  | Luhya | 0.0017 |
|  | **Total African** | **0.0055** |
|  | Kinh, Lahu, Cambodian, She, Miao, Tujia, Dai, Han | 0.0014 |
| East Asia | Japanese, Hezhen, Daur, Oroqen, Yakut, Xibo, Mongola, Yi, Tu, Naxi, Uygur | 0.0020 |
|  | **Total East Asian** | **0.0034** |
|  | Telegu, Tamil, Punjabi, Bengali, Gujarati, Burusho, Pathan, Sindhi | 0.0024 |
| Central and South Asia | Kalash, Brahui, Balochi, Makrani, Hazara | 0.0137 |
|  | **Total Central Asian** | **0.0160** |
| Oceania | Melanesian, Papuan | 0.0013 |
|  | **Total Oceanian** | **0.0013** |

**Supplementary Table 2. List of samples included in the panel for local ancestry**. African (AFR), Admixed Latino (AMR), or European (EUR) genotypes were obtained from the 1000 Genomes Project (1KGP), the Human Genome Diversity Project (HGDP), and from the GOCS cohort.

| AFR | HG02461, HG02463, HG02466, HG02575, HG02584, HG02585, HG02587, HG02590, HG02596, HG02611, HG02612, HG02615, HG02623, HG02624, HG02625, HG02644, HG02645, HG02647, HG02666, HG02667, HG02668, HG02677, HG02680, HG02704, HG02763, HG02764, HG02806, HG02809, HG02813, HG02815, HG02821, HG02837, HG02838, HG02839, HG02841, HG02852, HG02855, HG02856, HG02861, HG02862, HG02871, HG02878, HG02879, HG02880, HG02883, HG02886, HG02887, HG02889, HG02895, HG02896, HG02897, HG02922, HG02923, HG02924, HG02943, HG02944, HG02945, HG02948, HG02952, HG02953, HG02954, HG02964, HG02966, HG02971, HG02972, HG02974, HG02975, HG02977, HG02978, HG02980, HG02984, HG03029, HG03035, HG03047, HG03049, HG03050, HG03099, HG03100, HG03101, HG03108, HG03109, HG03110, HG03112, HG03113, HG03114, HG03115, HG03116, HG03118, HG03119, HG03121, HG03122, HG03123, HG03125, HG03127, HG03128, HG03129, HG03130, HG03131, HG03132, HG03133, HG03134, HG03136, HG03137, HG03159, HG03160, HG03161, HG03162, HG03163, HG03164, HG03166, HG03168, HG03169, HG03170, HG03175, HG03190, HG03191, HG03193, HG03195, HG03196, HG03197, HG03198, HG03199, HG03200, HG03242, HG03248, HG03260, HG03267, HG03268, HG03269, HG03270, HG03271, HG03272, HG03294, HG03295, HG03296, HG03297, HG03298, HG03299, HG03301, HG03302, HG03303, HG03304, HG03305, HG03306, HG03307, HG03308, HG03309, HG03310, HG03311, HG03313, HG03314, HG03342, HG03343, HG03344, HG03350, HG03351, HG03352, HG03366, HG03367, HG03368, HG03370, HG03371, HG03372, HG03373, HG03374, HG03499, HG03514, HG03515, HG03516, HG03517, HG03518, HG03519, HG03520, HG03521, HG03522, HG03540, HGDP00450, HGDP00452, HGDP00462, HGDP00463, HGDP00467, HGDP00468, HGDP00471, HGDP00472, HGDP00478, HGDP00981, HGDP00983, HGDP00984, HGDP01081, HGDP01087, HGDP01092, LP6005441-DNA-A08, LP6005441-DNA-B08, LP6005592-DNA-C03, NA18488, NA18497, NA18500, NA18502, NA18503, NA18504, NA18505, NA18854, NA18863, NA18870, NA18872, NA18909, NA18911, NA18914, NA18923, NA18925, NA18935, NA19094, NA19095, NA19100, NA19107, NA19116, NA19119, NA19120, NA19142, NA19148, NA19151, NA19161, NA19173, NA19186, NA19191, NA19197, NA19199, NA19202, NA19206, NA19208, NA19210, NA19211, NA19215, NA19221, NA19224, NA19238, NA19240, NA19258, SS6004471 |
| --- | --- |
| AMR | GOCS-18, GOCS-64, GOCS-203, GOCS-204, GOCS-238, GOCS-246, GOCS-282, GOCS-380, GOCS-508, GOCS-591, GOCS-653, GOCS-679, GOCS-706, GOCS-808, GOCS-1029, GOCS-1046, HG01572, HG01917, HG01919, HG01920, HG01922, HG01923, HG01926, HG01927, HG01928, HG01938, HG01940, HG01942, HG01943, HG01951, HG01952, HG01953, HG01954, HG01955, HG01961, HG01968, HG01974, HG01975, HG01992, HG01993, HG01997, HG01998, HG02004, HG02008, HG02104, HG02105, HG02106, HG02146, HG02147, HG02148, HG02150, HG02259, HG02261, HG02265, HG02266, HG02271, HG02272, HG02273, HG02275, HG02278, HG02291, HG02292, HG02293, HG02299, HGDP00704, HGDP00708, HGDP00710, HGDP00832, HGDP00837, HGDP00838, HGDP00843, HGDP00845, HGDP00849, HGDP00856, HGDP00858, HGDP00864, HGDP00865, HGDP00872, HGDP00873, HGDP00970, HGDP00995, HGDP00999, HGDP01001, HGDP01006, HGDP01009, HGDP01010, HGDP01013, HGDP01014, HGDP01019, HGDP01037, HGDP01041, HGDP01043, HGDP01050, HGDP01051, HGDP01053, HGDP01055, HGDP01056, HGDP01057, HGDP01059, HGDP01060, LP6005441-DNA-A04, LP6005441-DNA-A12, LP6005441-DNA-B04, LP6005441-DNA-B12, LP6005441-DNA-E10, LP6005441-DNA-F10, LP6005441-DNA-G06, LP6005441-DNA-G07, LP6005441-DNA-H06, LP6005441-DNA-H07, SS6004476 |
| EUR | HG00109, HG00110, HG00111, HG00120, HG00122, HG00126, HG00139, HG00146, HG00149, HG00150, HG00157, HG00174, HG00188, HG00235, HG00236, HG00252, HG00253, HG00260, HG00265, HG00268, HG00269, HG00273, HG00274, HG00275, HG00278, HG00280, HG00304, HG00310, HG00311, HG00318, HG00328, HG00329, HG00334, HG00335, HG00337, HG00338, HG00345, HG00346, HG00350, HG00351, HG00357, HG00376, HG00379, HG00380, HG00381, HG00383, HG01500, HG01501, HG01502, HG01503, HG01504, HG01505, HG01506, HG01507, HG01508, HG01509, HG01510, HG01511, HG01512, HG01513, HG01514, HG01515, HG01516, HG01517, HG01518, HG01519, HG01520, HG01521, HG01522, HG01523, HG01524, HG01525, HG01526, HG01528, HG01529, HG01531, HG01532, HG01536, HG01537, HG01538, HG01601, HG01603, HG01604, HG01605, HG01606, HG01608, HG01609, HG01610, HG01611, HG01612, HG01617, HG01622, HG01623, HG01629, HG01671, HG01672, HG01673, HG01674, HG01675, HG01676, HG01677, HG01678, HG01679, HG01680, HG01681, HG01682, HG01684, HG01686, HG01687, HG01698, HG01700, HG01701, HG01702, HG01703, HG01704, HG01705, HG01706, HG01707, HG01709, HG01710, HG01711, HG01747, HG01748, HG01761, HG01763, HG01765, HG01766, HG01767, HG01770, HG01771, HG01772, HG01773, HG01774, HG01775, HG01776, HG01777, HG01778, HG01779, HG01780, HG01782, HG01783, HG01784, HG01785, HG01786, HG02219, HG02221, HG02222, HG02223, HG02224, HG02225, HG02230, HG02231, HG02232, HG02233, HG02234, HG02235, HG02238, HGDP00517, HGDP00523, HGDP00531, HGDP00534, HGDP00538, HGDP00607, HGDP00610, HGDP00612, HGDP00618, HGDP00623, HGDP00624, HGDP00630, HGDP00631, HGDP00636, HGDP00641, HGDP00648, HGDP00649, HGDP00651, HGDP00653, HGDP00666, HGDP00667, HGDP00668, HGDP00669, HGDP00670, HGDP00671, HGDP00672, HGDP00673, HGDP00674, HGDP00701, HGDP00802, HGDP00806, HGDP00810, HGDP01062, HGDP01063, HGDP01065, HGDP01066, HGDP01068, HGDP01069, HGDP01070, HGDP01071, HGDP01072, HGDP01073, HGDP01074, HGDP01075, HGDP01077, HGDP01357, HGDP01358, HGDP01359, HGDP01360, HGDP01361, HGDP01362, HGDP01363, HGDP01366, HGDP01367, HGDP01369, HGDP01370, HGDP01371, HGDP01372, HGDP01373, HGDP01374, HGDP01375, HGDP01376, HGDP01377, HGDP01378, HGDP01379, HGDP01380, LP6005441-DNA-C02, LP6005441-DNA-C11, LP6005441-DNA-D02, LP6005441-DNA-D11, LP6005441-DNA-E02, LP6005441-DNA-F02, NA06984, NA06986, NA06989, NA06993, NA06995, NA06997, NA07000, NA07019, NA07022, NA07029, NA07045, NA07048, NA07051, NA07055, NA07056, NA07345, NA07346, NA07349, NA10830, NA10831, NA10835, NA10836, NA10839, NA10846, NA10847, NA10851, NA10852, NA10856, NA10857, NA10859, NA10860, NA10861, NA10863, NA10864, NA10865, NA11829, NA11830, NA11840, NA11882, NA11894, NA11932, NA11993, NA11995, NA12003, NA12005, NA12006, NA12044, NA12045, NA12046, NA12056, NA12057, NA12145, NA12146, NA12155, NA12234, NA12236, NA12248, NA12275, NA12329, NA12336, NA12342, NA12344, NA12376, NA12386, NA12399, NA12400, NA12413, NA12414, NA12485, NA12489, NA12707, NA12716, NA12739, NA12748, NA12762, NA12766, NA12767, NA12776, NA12777, NA12778, NA12802, NA12814, NA12815, NA12817, NA12818, NA12827, NA12828, NA12829, NA12830A, NA12832, NA12875, NA12877, NA12878, NA12890, NA12891, NA12892, SS6004474 |

**Supplementary Table 3. The average proportion of global ancestry for the 676 samples included in the panel for local ancestry**. Global ancestry is expressed in the Region and Population-level, including admixed Latino (AMR), European (EUR), African (AFR), East Asian (EAS), South Asian (SAS), and Oceanic (OCE) ancestry.

| **Region** | **Population** | **Project** | **Samples** | **NAT** | **EUR** | **AFR** | **EAS** | **SAS** | **OCE** |
| --- | --- | --- | --- | --- | --- | --- | --- | --- | --- |
|  | Probably Mapuche | GOCS | 16 | 0.9966 | 0.0022 | 0.0000 | 0.0000 | 0.0010 | 0.0002 |
|  | Colombian | HGDP | 6 | 0.9999 | 0.0000 | 0.0000 | 0.0000 | 0.0000 | 0.0000 |
|  | Karitiana | HGDP | 12 | 0.9999 | 0.0000 | 0.0000 | 0.0000 | 0.0000 | 0.0000 |
|  | Maya | HGDP | 8 | 0.9854 | 0.0000 | 0.0000 | 0.0142 | 0.0000 | 0.0003 |
| AMR | Peruvian | 1KGP | 48 | 0.9984 | 0.0013 | 0.0000 | 0.0002 | 0.0000 | 0.0000 |
|  | Pima | HGDP | 13 | 0.9916 | 0.0000 | 0.0000 | 0.0083 | 0.0000 | 0.0000 |
|  | Surui | HGDP | 8 | 0.9999 | 0.0000 | 0.0000 | 0.0000 | 0.0000 | 0.0000 |
|  | **Total American** | | **111** | **0.9967** | 0.0009 | 0.0000 | 0.0021 | 0.0002 | 0.0001 |
|  | Basque | HGDP | 23 | 0.0000 | 0.9987 | 0.0000 | 0.0000 | 0.0008 | 0.0005 |
|  | Bedouin | HGDP | 17 | 0.0000 | 0.9961 | 0.0025 | 0.0000 | 0.0003 | 0.0010 |
|  | Northern and Western Europe | 1KGP | 96 | 0.0000 | 0.9850 | 0.0000 | 0.0000 | 0.0148 | 0.0000 |
|  | Finnish | 1KGP | 29 | 0.0000 | 0.9767 | 0.0000 | 0.0221 | 0.0009 | 0.0003 |
| EUR | French | HGDP | 5 | 0.0000 | 0.9780 | 0.0000 | 0.0000 | 0.0216 | 0.0003 |
|  | British | 1KGP | 17 | 0.0000 | 0.9758 | 0.0000 | 0.0000 | 0.0241 | 0.0000 |
|  | Iberian | 1KGP | 111 | 0.0000 | 0.9861 | 0.0014 | 0.0001 | 0.0116 | 0.0007 |
|  | Orcadian | HGDP | 3 | 0.0000 | 0.9732 | 0.0000 | 0.0000 | 0.0267 | 0.0000 |
|  | Sardinian | HGDP | 25 | 0.0000 | 0.9998 | 0.0000 | 0.0000 | 0.0000 | 0.0001 |
|  | **Total European** | | **326** | 0.0000 | **0.9866** | 0.0006 | 0.0020 | 0.0103 | 0.0004 |
|  | Esan | 1KGP | 115 | 0.0000 | 0.0000 | 0.9999 | 0.0000 | 0.0000 | 0.0000 |
|  | Gambian | 1KGP | 61 | 0.0000 | 0.0000 | 0.9999 | 0.0000 | 0.0000 | 0.0000 |
| AFR | Pygmy | HGDP | 19 | 0.0000 | 0.0000 | 0.9999 | 0.0000 | 0.0000 | 0.0000 |
|  | Yoruba | 1KGP | 44 | 0.0000 | 0.0000 | 0.9999 | 0.0000 | 0.0000 | 0.0000 |
|  | **Total African** | | **239** | 0.0000 | 0.0000 | **0.9999** | 0.0000 | 0.0000 | 0.0000 |

**Supplementary Table 4. Comparison in the proportion of ancestry estimated with ADMIXTURE or RFMix**. Values for the 16 GOCS samples included in the reference panel for local ancestry are shown. The average values for the full GOCS cohort estimated with each methodology are at the bottom of the table.

|  | **Native American** | | **European** | | **African** | |
| --- | --- | --- | --- | --- | --- | --- |
| GOCS Samples | ADMIXTURE | RFMix | ADMIXTURE | RFMix | ADMIXTURE | RFMix |
| 1 | 0.99988 | 0.95915 | 0.00003 | 0.03992 | 0.00004 | 0.00090 |
| 2 | 0.99988 | 0.94552 | 0.00003 | 0.05271 | 0.00004 | 0.00175 |
| 3 | 0.99988 | 0.90801 | 0.00003 | 0.08942 | 0.00004 | 0.00255 |
| 4 | 0.99988 | 0.89683 | 0.00003 | 0.10061 | 0.00004 | 0.00253 |
| 5 | 0.99988 | 0.85810 | 0.00003 | 0.13820 | 0.00004 | 0.00367 |
| 6 | 0.99988 | 0.85028 | 0.00003 | 0.14365 | 0.00004 | 0.00604 |
| 7 | 0.99988 | 0.84904 | 0.00003 | 0.13579 | 0.00004 | 0.01514 |
| 8 | 0.99988 | 0.80177 | 0.00003 | 0.19304 | 0.00004 | 0.00516 |
| 9 | 0.99988 | 0.79812 | 0.00003 | 0.19597 | 0.00004 | 0.00589 |
| 10 | 0.99988 | 0.79347 | 0.00003 | 0.19243 | 0.00004 | 0.01407 |
| 11 | 0.99988 | 0.77987 | 0.00003 | 0.21165 | 0.00004 | 0.00845 |
| 12 | 0.99963 | 0.76274 | 0.00028 | 0.23111 | 0.00004 | 0.00613 |
| 13 | 0.99202 | 0.77882 | 0.00789 | 0.20548 | 0.00004 | 0.01567 |
| 14 | 0.99196 | 0.77497 | 0.00795 | 0.21957 | 0.00004 | 0.00544 |
| 15 | 0.98966 | 0.76697 | 0.01025 | 0.21562 | 0.00004 | 0.01738 |
| 16 | 0.97302 | 0.74618 | 0.00809 | 0.23702 | 0.00004 | 0.01678 |
| **Average ancestry** | **0.99656** | **0.82936** | **0.00217** | **0.16264** | **0.00004** | **0.00797** |
| **Average in the full GOCS cohort** | **0.6135** | **0.4561** | **0.3603** | **0.5248** | **0.0055** | **0.0190** |

**Supplementary Table 5. Minor allele frequency for top variants in the full GOCS cohort, and in the LAD-GWAS for Native American and European ancestry.** The frequencies described for genomes of the gnomAD project for the admixed Latino population (AMR) and for the European non-Finnish population (EUR) are also included. Also shown is the frequency of variants with Native American (NAT) ancestry estimated with a similar approximation in gnomAD and the frequency from the HGDP project (includes Maya, Colombian, Karitiana, Pima and Surui). In bold the most relevant differences in frequency between the NAT and EUR ancestry are highlighted.

| **Variant** | **Ref** | **Alt** | **MAF Cohort** | **MAF NAT** | **MAF EUR** | **MAF gnomAD Admixed AMR** | **MAF gnomAD EUR** | **MAF gnomAD NAT** | **MAF HGDP NAT** |
| --- | --- | --- | --- | --- | --- | --- | --- | --- | --- |
| ***Full GOCS Cohort GWAS*** | | | | | | | | | |
| rs1551285 | A | C | 0.769 | 0.701 | 0.799 | 0.812 | 0.831 | - | 0.729 |
| **rs28969691** | **T** | **C** | **0.102** | **0.198** | **0.009** | **0.062** | **0.002** | **0.243** | **0.199** |
| rs887829 | C | T | 0.325 | 0.342 | 0.299 | 0.324 | 0.321 | 0.371 | 0.270 |
| rs10169532 | C | T | 0.393 | 0.434 | 0.337 | 0.378 | 0.396 | - | 0.543 |
| rs17868361 | G | A | 0.134 | 0.134 | 0.138 | 0.145 | 0.164 | 0.190 | 0.132 |
| **rs116206753** | **G** | **A** | **0.057** | **0.108** | **0.022** | **0.053** | **0.012** | **0.157** | **0.109** |
| **rs76776133** | **T** | **A** | **0.372** | **0.517** | **0.207** | **0.292** | **0.229** | **-** | **0.372** |
| **rs10900821** | **C** | **T** | **0.090** | **0.007** | **0.147** | **0.123** | **0.163** | **-** | **0.000** |
| **rs1646747** | **G** | **A** | **0.173** | **0.025** | **0.283** | **0.192** | **0.261** | **-** | **0.044** |
| **rs34437032** | **G** | **A** | **0.267** | **0.381** | **0.151** | **0.210** | **0.160** | **0.343** | **0.410** |
| **rs112547984** | **G** | **A** | **0.071** | **0.010** | **0.108** | **0.070** | **0.091** | **-** | **0.014** |
| **rs1910167** | **T** | **C** | **0.083** | **0.003** | **0.142** | **0.104** | **0.129** | **0.029** | **0.0002** |
| **rs455868** | **G** | **A** | **0.766** | **0.686** | **0.853** | **0.773** | **0.808** | **-** | **0.557** |
| **rs34426376** | **C** | **T** | **0.107** | **0.006** | **0.196** | **0.132** | **0.161** | **-** | **0.014** |
| **rs4810066** | **T** | **C** | **0.796** | **0.961** | **0.661** | **0.762** | **0.674** | **-** | **0.971** |
| rs76031163 | C | G | 0.132 | 0.178 | 0.114 | 0.094 | 0.079 | 0.129 | 0.120 |
| ***NAT local ancestry deconvoluted GWAS*** | | | | | | | | | |
| rs887829 | C | T | 0.325 | 0.342 | 0.299 | 0.324 | 0.321 | 0.371 | 0.270 |
| rs9813423 | G | A | 0.227 | 0.240 | 0.197 | 0.228 | 0.242 | - | 0.257 |
| **rs79896759** | **A** | **G** | **0.080** | **0.140** | **0.045** | **0.038** | **0.005** | **0.086** | **0.070** |
| rs6962785 | T | C | 0.779 | 0.795 | 0.753 | 0.781 | 0.728 | - | 0.800 |
| rs1359822 | T | C | 0.761 | 0.837 | 0.690 | 0.731 | 0.719 | - | 0.814 |
| rs536866 | A | G | 0.200 | 0.223 | 0.180 | 0.198 | 0.178 | 0.314 | 0.221 |
| rs4627235 | G | A | 0.267 | 0.266 | 0.243 | 0.287 | 0.246 | 0.221 | 0.302 |
| ***EUR local ancestry deconvoluted GWAS*** | | | | | | | | | |
| rs887829 | C | T | 0.325 | 0.342 | 0.299 | 0.324 | 0.321 | 0.371 | 0.270 |
| rs14576 | C | A | 0.317 | 0.261 | 0.389 | 0.371 | 0.352 | 0.300 | 0.300 |
| rs13147930 | C | T | 0.113 | 0.108 | 0.128 | 0.120 | 0.081 | - | 0.143 |
| **rs10900821** | **C** | **T** | **0.090** | **0.007** | **0.147** | **0.123** | **0.163** | **-** | **0.000** |
| **rs1592334** | **G** | **A** | **0.124** | **0.089** | **0.138** | **0.142** | **0.151** | **0.057** | **0.092** |
| **rs41288897** | **T** | **A** | **0.361** | **0.639** | **0.209** | **0.346** | **0.225** | **0.743** | **0.711** |
| **rs1107560** | **G** | **T** | **0.224** | **0.383** | **0.091** | **0.137** | **0.092** | **0.229** | **0.211** |
| **rs71523681** | **T** | **G** | **0.069** | **0.003** | **0.133** | **0.080** | **0.144** | **0.014** | **0.002** |
| **rs4837851** | **T** | **C** | **0.746** | **0.528** | **0.917** | **0.840** | **0.897** | **-** | **0.757** |
| rs12414558 | G | A | 0.101 | 0.106 | 0.097 | 0.115 | 0.102 | - | 0.071 |
| rs2606106 | A | C | 0.378 | 0.346 | 0.393 | 0.339 | 0.422 | - | 0.257 |
| **rs4391796** | **T** | **C** | **0.313** | **0.383** | **0.213** | **0.357** | **0.199** | **-** | **0.500** |
| **rs1910167** | **T** | **C** | **0.083** | **0.003** | **0.142** | **0.104** | **0.129** | **0.029** | **0.0002** |
| rs2695289 | G | T | 0.746 | 0.722 | 0.750 | 0.803 | 0.762 | 0.871 | 0.789 |
| **rs12901092** | **C** | **A** | **0.318** | **0.186** | **0.463** | **0.314** | **0.414** | **-** | **0.186** |
| rs76335095 | G | T | 0.058 | 0.052 | 0.053 | 0.072 | 0.046 | 0.086 | 0.072 |
